# A Highlight of Recent Advances in Aptamer Technology and Its Application

**DOI:** 10.3390/molecules200711959

**Published:** 2015-06-30

**Authors:** Hongguang Sun, Youli Zu

**Affiliations:** Department of Pathology and Genomic Medicine, Houston Methodist Hospital, Houston, TX 77030, USA; E-Mail: sunhongguang78@aliyun.com

**Keywords:** aptamer, SELEX, modification, optimization, improvement, application

## Abstract

Aptamers and SELEX (systematic evolution of ligands by exponential enrichment) technology have gained increasing attention over the past 25 years. Despite their functional similarity to protein antibodies, oligonucleotide aptamers have many unique properties that are suitable for clinical applications and industrialization. Aptamers may be superior to antibodies in fields such as biomarker discovery, *in vitro* and *in vivo* diagnosis, precisely controlled drug release, and targeted therapy. However, aptamer commercialization has not occurred as quickly as expected, and few aptamer-based products have yet successfully entered clinical and industrial use. Thus, it is important to critically review some technical barriers of aptamer and SELEX technology *per se* that may impede aptamer development and application. To date, how to rapidly obtain aptamers with superior bioavailability over antibodies remains the key issue. In this review, we discuss different chemical and structural modification strategies aimed to enhance aptamer bioavailability. We also discuss improvements to SELEX process steps to shorten the selection period and improve the SELEX process success rate. Applications in which aptamers are particularly suited and perform differently or superior to antibodies are briefly introduced.

## 1. Introduction

**Figure 1:**
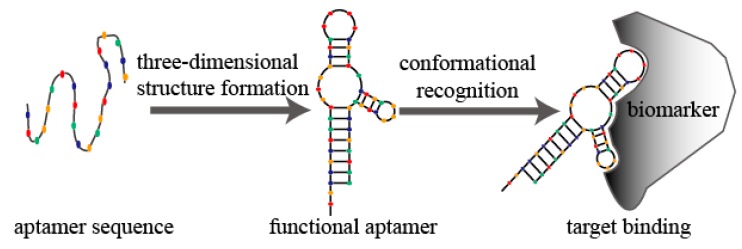
Schematic diagram of aptamer conformational recognition of targets to form an aptamer-target complex.

In the past 25 years, aptamers and SELEX (systematic evolution of ligands by exponential enrichment) technology have gained great attention [1,2]. Aptamers are essentially short RNA or single-stranded DNA oligonucleotides (usually 20–80 nucleotides with 6–30 kDa molecular weights) that can fold into unique three-dimensional conformations. Similar to conformational recognition that mediates antibody–antigen recognition and complex formation, aptamers bind to their cognate targets using high-specificity and -affinity through van der Waals forces, hydrogen bonding, electrostatic interactions, stacking of flat moieties, and shape complementarity (Figure 1) [3,4], with dissociation constants (*Kd*) usually ranging from pico- to nanomolar [5]. Thus, aptamers are also referred to as “chemical antibodies” and are functionally used as antagonists, agonists, or targeting ligands [6,7].

**Figure 2:**
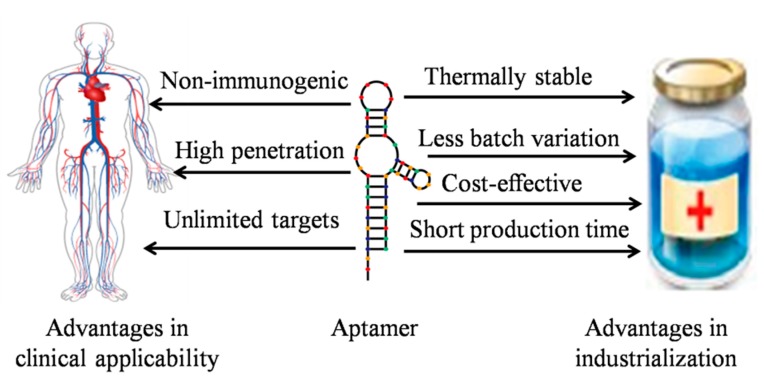
Important advantages of aptamers over antibodies in clinical applicability and industrialization.

Aptamers are excellent alternatives or supplements to monoclonal antibodies, which suffer from high immunogenicity and production costs. Compared to antibodies, aptamers offer several unambiguous advantages due to their smaller size and nucleic acid characteristics that can improve their clinical applicability and suitability for industrialization (Figure 2). Aptamers have superior clinical applicability over antibodies in several respects: (i) Aptamers are virtually nonimmunogenic and nontoxic *in vivo* [8,9]. One important reason is that oligonucleotide aptamers are not directly recognized by the immune system. Aptamers also do not have redundant Fc regions like antibodies that can bind to Fc receptors and lead to unforeseen side effects [10]; (ii) Due to their smaller size, aptamers can robustly penetrate into tissue barriers and be easily internalized by their target cells, thus improving tumor-to-blood and tumor-to-normal tissue ratios and enhancing their therapeutic indices [11]; (iii) Aptamers can be developed against a seemingly unlimited range of targets. To date, specific aptamers against diverse targets have been successfully developed, including small inorganic ions, drugs, organic peptides, proteins, and even complex cells or tissues [12,13,14,15,16,17], which greatly expands the scope of aptamer applications. Furthermore, aptamers have important properties that simplify their industrialization; (iv) Aptamers are thermally stable, so they can be stored and transported easily; (v) Based on well-established chemical synthesis and modifications technologies, a given aptamer can be produced or modified in large scale, with minimal batch-to-batch variation and in a short time (hours).

**Figure 3:**
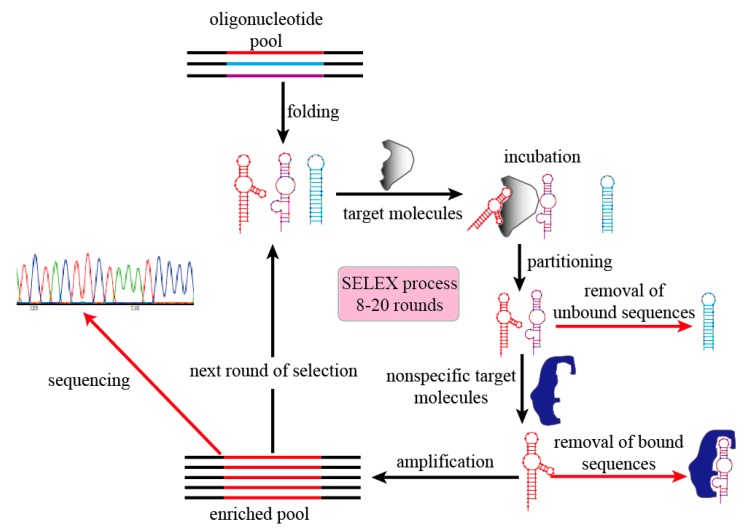
Schematic diagram of the SELEX process steps.

SELEX is the gold-standard methodology for developing specific aptamers. In principle, the conventional SELEX process includes multiple rounds of exponential amplification and enrichment, which allows evolution of aptamers with high target-specific affinity from a random oligonucleotide pool [1,2]. The SELEX process usually contains several steps to successfully generate target-specific aptamers (Figure 3). (i) Preparation of initial oligonucleotide pool: a single-stranded DNA oligonucleotide pool comprised of 10^14^–10^15^ random sequences is first carefully designed and chemically synthesized. Each unique sequence usually contains random oligonucleotides (30–50 nt) between two conserved primer binding sites; (ii) Incubation: random sequences in the initial pool will fold into different secondary and tertiary structures, which are then incubated with immobilized or free targets under optimal conditions to form aptamer-target complexes; (iii) Partitioning: unbound sequences are separated from target-bound sequences through different methods, such as membrane filtration, affinity columns, magnetic beads, or capillary electrophoresis [18]; (iv) Amplification: target-bound sequences are amplified by PCR (DNA aptamers) or RT-PCR (RNA aptamers), and reaction products are used as a new aptamer sub-pool for the next selection round; (v) Sequencing: enriched aptamer sequences are identified by classic Sanger sequencing or high-throughput sequencing methods. Usually, several negative-target selections (counter-selections) are added to the process that can eliminate nonspecific sequences generated by their binding to non-target moieties. Specific aptamers can be obtained after 8–20 rounds of selection, with the entire process taking weeks to months.

Although the conventional SELEX technique and dozens of variations have been used successfully to develop aptamers against hundreds of targets, including synthetic peptides or purified proteins [18,19,20], aptamers developed through these types of SELEX techniques are not always effective for *in vivo* applications [21]. This is because target molecule conformations during *in vitro* selection are usually different from their native conformations under *in vivo* physiological conditions, and conformational recognition is the most important parameter for aptamer binding to their cognate targets. To close this gap, a modified SELEX technology that uses whole living cells as targets, Cell-SELEX, was established recently [22,23,24,25]. Data to date support that aptamers generated by Cell-SELEX have excellent clinical applicability.

As of June 2015, there were >5000 published articles in the PubMed database that included the term “aptamer”. Research fields that include aptamer technology include biosensors (aptasensors), bioimaging probes, nanotechnology, targeted diagnosis, gene therapy, and many more. However, aptamer commercialization has not been achieved as quickly as originally expected, and few successful aptamer-based products exist in the clinic or markets after 25 years of development [26]. So far, only one aptamer-based drug, Macugen (pegaptanib sodium, Pfizer/Eyetech), an RNA aptamer specific against vascular endothelial growth factor (VEGF), was approved by the US Food and Drug Administration (FDA) in 2004 for treating wet age-related macular degeneration [27]. Ten other therapeutic aptamers are being evaluated in clinical trials for their effectiveness and safety in treating macular degeneration and other disorders involving coagulation, oncology, and inflammation [10,28].

With 25 years of rapid development, it is important to carefully review some technical barriers of aptamer and SELEX technology, whose correction could accelerate aptamer development for clinical applications and commercialization. There are two main barriers that severely impede development and application of aptamers. The first impediment is that nucleic acid aptamers generated *in vitro* have different bioavailability and binding characteristics for *in vivo* applications, and the second hindrance is that the SELEX process is usually time-consuming and suffers from low success rates. Thus, the most important issue up to now is how to rapidly obtain superior bioavailability of aptamers. This review will discuss the different strategies aimed at enhancing aptamer bioavailability, shortening the selection period, and improving the success rate of the SELEX process. These include approaches such as aptamer modification by incorporating modified nucleotides and/or conjugation with bioavailable nanomaterials and improving the SELEX process by optimizing oligonucleotide pool design and improving amplification and sequencing methods. Additionally aptamer applications, especially in models different from or superior to those of monoclonal antibodies, are briefly introduced.

## 2. Aptamer Modifications and Improved Strategies in SELEX Technology

### 2.1. Aptamer Modifications

Being a class of small nucleic acids, high nuclease-sensitivity and fast renal filtration are two main technical problems of aptamers that result in suboptimal bioavailability for *in vivo* applications [29]. To improve aptamer bioavailability, strategies have been developed to chemically modify backbone or side chains, incorporate unnatural nucleotides, and cap aptamer ends [11]. Moreover, utilization of these improved strategies will provide several additional advantages, including improved aptamer affinity and increased library diversity, which improve the success rate of SELEX [30].

Structurally, most ribonucleases polarize the 2′-OH group to attack the phosphodiester linkage leading to hydrolysis of RNA sequences. Subsequently, chemical substitution of RNA 2′-OH moieties with different chemical groups (e.g., 2′-fluoro, 2′-amino, or 2′-*O*-methoxy motifs) [31,32,33] and/or modifying the native phosphodiester backbone with boranophosphate or phosphorothioate residues [30,34] are the most common strategies to improve aptamer ribonuclease resistance. Some approaches perform both modifications simultaneously, e.g., substitutions with phosphorodithioate and 2′-*O*-methyl in one nucleotide [35]. Also, aptamers modified by 2′-*O*-methoxy group achieve enhanced clinical safety [8,9]. Although evidence to date in preclinical and clinical trials show that aptamers are nonimmunogenic and nontoxic, scientists still worry that aptamers may be detected by Toll-like receptors to induce unwanted immune responses. However, RNA molecules modified by 2′-*O*-methoxy cannot activate Toll-like receptor signaling [36]. Some other notable modification strategies have also shown their effectiveness to improve ribonuclease resistance and enhance affinity of RNA aptamers, such as the incorporation of locked nucleic acids (LNAs) [37] or XNA technology [38] and the generation of “mirror” structures of aptamers named Spiegelmers (NOXXON Pharma AG, which has three Spiegelmers-based products being evaluated under clinical trials) [39]. Although DNA aptamers are more stable than RNA aptamers [40] due to the lack of 2′-OH groups, incorporating either LNAs or phosphorothioate backbones can also improve the biostability of DNA aptamers [21,41].

Another innovative method to improve aptamer affinity, nuclease-resistance, and the success rate of SELEX is the Slow Off-rate Modified Aptamer (SOMAmer^®^, SomaLogic Inc., Boulder, CO, USA) technology. Though traditional SELEX can be theoretically used to select high-affinity aptamers against various targets, the practical success rate is usually <30% [20]. The reasons for this technical barrier may include: (i) the conformational diversity of nucleic acids is limited more than that of monoclonal antibodies and (ii) nucleic acid aptamers with highly negative charges have difficulty binding with the negatively charged target molecules [10]. Inspired by the idea that the chemical diversity of proteins can be expanded by adding functional groups to amino acid side-chains [42], Gold and colleagues developed a random aptamer library comprised of 5′-position modified deoxyuridines that expanded library diversity. Importantly, this modification also improves aptamer-binding affinity by forming additional hydrophobic interactions between aptamers and their cognate targets that are superior to the conventional binding force (e.g., hydrogen bonds) [20,43]. In a seminal experiment, aptamers (with *Kd* < 30 nM) against >1000 targets, including some “difficult” proteins that failed to select from an unmodified library, were generated with an improved total success rate of approximately 84% [20]. With the success of SOMAmer technology in high-throughput protein selection, SomaLogic Inc. developed a SOMAScan aptamer array platform that is used in proteomics and diagnostics markets that has a current analytic detection limit in the femtomolar range for over 1100 proteins in small volumes of body fluids (150 μL of serum or plasma) [26,44]. Kimoto *et al*., similarly developed a modified oligonucleotide pool by incorporating an unnatural nucleotide with the hydrophobic base 7-(2-thienyl)imidazo[4,5-*b*]pyridine, and successfully generated aptamers against VEGF and interferon-γ [45]. Their data showed significantly improved binding affinity of the resultant aptamers (>500 fold) *vs.* unmodified aptamers, with dissociation constants ranging between low picomolar to high femtomolar.

In consideration of the problem involving fast renal filtration, the effective circumventing strategy is to conjugate aptamers with large bioavailable nanomaterials, such as polyethylene glycol (PEG), that increase the total molecular weight of the resultant aptamer-nanomaterial complex beyond the renal filtration threshold of 40 kDa [46]. PEG modification, also termed PEGylation, is the most common and safe strategy used in small-drug modification. Data to date have shown that PEGylated aptamers not only have increased blood residence time, but can also enhance their nuclease-resistance and decrease their toxic accumulation in non-target tissues [47,48]. Currently, the most successful PEGylated aptamer is Macugen, the first FDA-approved aptamer-based drug. After incorporating 2′-fluoro pyrimidine, 2′-*O*-methyl purines and an inverted nucleotide at the 3′-terminus, a 40 kDa PEG molecule was conjugated to the RNA sequence. Derived from combinations of these modifications, Macugen exhibits prolonged blood residence with a mean apparent terminal half-life of up to 10 days in humans. Additionally, no unwanted immune response was detected [49,50].

In addition to PEG polymers, other bioavailable nanomaterials are used to modify aptamers for reducing their renal clearance, such as gold nanoparticles, liposomes, and copolymers. Intriguingly, aptamers conjugated with nanomaterials can have some additional advantages, such as enhanced nuclease resistance and increased affinity due to multivalent effects. For example, due to strong steric effects and high ionic charge, gold nanoparticles can effectively stabilize aptamers. When specific DNA aptamers against human HIV-1 reverse transcriptase were conjugated with 13-nm gold nanoparticles, the aptamers showed significantly improved nuclease resistance, even in DNase-containing buffer [51]. In another experiment, the TD05 aptamer against the immunoglobulin mu heavy chain overexpressed on B-cell lymphoma cells had high affinity at 4 °C, but lost its affinity at 37 °C [52]. However, when TD05 aptamers were conjugated with a lipid-tail and formed into aptamer-micelles, the multivalent effects improved their binding affinity approximately 750-fold, even at 37 °C.

In summary, utilizing one or a combination of several kinds of aptamer-modification strategies significantly improves aptamer biostability and bioavailability. Indeed, some modified aptamers in preclinical or clinical stages exemplify the effectiveness of these modification strategies. Nevertheless, for a specific aptamer, there is no fixed pattern to direct selection of the best modification strategy. Some modification strategies will negatively influence the interaction between an aptamer and its target. Thus, each aptamer modification should be carefully designed, optimized, and evaluated to achieve the desired outcomes.

### 2.2. Improved Strategies in SELEX Technology

The conventional SELEX process usually suffers from a low success rate and is time consuming. Newer improved strategies have been applied at each step of the SELEX process, including improving design of the oligonucleotide pool, partitioning, amplification, and sequencing. These modified techniques aim to shorten the selection period, improve success rate, and further improve aptamer binding affinity or special functionality.

#### 2.2.1. Design of Oligonucleotide Pool

As the first step of a successful SELEX process, carefully designing the chosen oligonucleotide pool is the most important issue. Several key parameters should be considered, such as the length of the random core region, incorporation of modified nucleotides, and the random type of oligonucleotide pool. In general, the length of the random core region ranges usually between 30 and 50 nucleotides, which is enough to generate specific aptamers for most targets. For some special target molecules, against which high-affinity and specificity aptamers are difficult to obtain, increasing the random core region length to 100–200 nucleotides is an effective strategy to overcome this problem. Nowadays, it is not a hard task to chemically synthesize lengthier oligonucleotides up to 200 bases in large scale, but it was difficult in the past. Longer random sequences can form more complicated three-dimensional structures and binding sites, which improves the aptamer pool diversity and the likelihood of generating high-affinity aptamers against difficult targets [53,54,55,56].

Another important parameter to consider is whether to incorporate modified nucleotides. Modified nucleotides provide several advantages, such as improved binding affinity, enhanced nuclease resistance, more diversity of the oligonucleotide pool, and improved success rate. Using a modified oligonucleotide pool is becoming more popular, especially for generating RNA-based aptamers. Nevertheless, modified nucleotides cannot be recognized by wild-type T7 RNA polymerase. Fortunately, modified nucleotides can be recognized by some mutant RNA polymerases (detailed description in [30]), such as Y639F T7 RNA polymerase for incorporating 2′-fluoro pyrimidines [33,57] and Y693F/H784A or R425C T7 RNA polymerases for incorporating 2′-*O*-methyl nucleotides [31,58]. However, the efficiency of mutant T7 RNA polymerase is somewhat low so the reaction conditions should be carefully optimized.

Usually, the random type of an oligonucleotide pool includes complete and partial (doped) randomization [59]. To select aptamers against totally new targets, a completely random oligonucleotide pool is chosen. However, there may be many aptamers obtained previously that have suboptimal affinity or specificity. Based on the critical motif whose structure is responsible for binding with the target molecule, a doped pool can be synthesized to re-select optimal aptamers, which can usually obtain high-affinity aptamers rapidly. For example, high-affinity aptamers against HIV-1 aspartyl protease were successfully obtained from a doped pool based on the structural information of two previously obtained aptamers, and their binding affinity was improved from 92–140 nM to 2–22 nM *Kds* [60]. In some special conditions, such as developing aptamers against intracellular targets, aptamers developed in selection buffers that differ from the target environment may lose binding affinity because the complicated intracellular ionic composition may change the three-dimensional structure of aptamers. Using a doped pool, Lennarz *et al*., re-selected functional high-affinity aptamers against intracellular Erk2 protein by changing the selection buffer to one more similar to the intracellular environment [61].

#### 2.2.2. Partitioning

Efficient separation of unbound sequences from target-bound aptamer sequences is the most crucial step, and optimization can shorten selection period significantly. To date, a plethora of techniques have been introduced in the SELEX process to improve separation efficiency, such as magnetic beads, affinity chromatography, capillary electrophoresis, and microfluidic technology [18,19]. These techniques can usually shorten the selection period to 4–8 rounds. In a recent study, Luo *et al*. utilized the high partitioning efficiency of capillary electrophoresis and combined it with a fraction collection approach in their SELEX process [62]. In only one round of selection, they obtained high-affinity aptamers against streptavidin. Another innovative technique, non-equilibrium capillary electrophoresis of equilibrium mixtures (NECEEM), has also been applied to SELEX [63]. In NECEEM approach, target molecules are firstly incubated with aptamer pool to reach equilibrium conditions, and then, the equilibrium mixtures including target/aptamer complexes and free molecules perform non-equelibrium capillary electrophoresis by pure separation buffer. With the different dissociation and migration rates between target/aptamer complexes and free molecules, the target-specific aptamers are separated from non-specific sequences. This approach cannot only generate high-affinity aptamers in only a few selection rounds, but it can also simultaneously determine the binding parameters of aptamer-target interactions (*i.e*., equilibrium dissociation constant (*Kd*) and rate constants of complex formation (*k_on_*) and dissociation (*k_off_*)). For more detailed descriptions of efficient partitioning, see references [18,19].

#### 2.2.3. Amplification

Theoretically, conventional PCR reactions can effectively amplify the target-bound sequences. However, this is not always true. Due to the complexity of an oligonucleotide pool containing a plethora of diverse random sequences as templates, conventional PCR amplification is usually inefficient and prone to produce more byproducts rather than specific aptamer sequences [64]. Although optimizing PCR reaction conditions (e.g., primer concentration, amplification cycles, and annealing temperature) can reduce the amount of non-specific sequences to some extent, it is not always effective. Thus, inefficient PCR amplification is another key barrier to SELEX success. To overcome this obstacle, an effective PCR method named emulsion PCR (ePCR), which can diffuse complex sequence templates to form single-sequence templates and thereby reducing byproduct formation significantly, was introduced to the SELEX amplification process. For example, by combining ePCR amplification and NECEEM partitioning, Yufa *et al*. successfully developed high-affinity aptamers against AlkB homologue 2, a DNA damage repair enzyme, in three rounds of selection, while SELEX with conventional PCR failed [65]. With the same aim of reducing byproducts in PCR reactions, a high-fidelity digital PCR platform was also recently developed. After three rounds of selection, specific aptamers against human α-thrombin were successfully generated [66]. In another study, by combining solid-phase ePCR amplification and magnetic separation, a semiautomated SELEX platform was developed. This platform can implement 12 different SELEX protocols simultaneously in 10 days [67].

With respect to DNA starting material for aptamer production, preparing sufficient single-stranded DNA from double-stranded DNA amplicons by alkaline denaturation and streptavidin-biotin affinity purification to generate a new sub-pool has inherent limitations. For example, alkaline denaturation has a low recovery rate, which will lose some binding sequences and potentially damage the three-dimensional structure of aptamers due to hydrolysis of side chain functional groups. Asymmetric PCR, which can produce sufficient single-stranded DNA with high efficiency and no damage to binding sequences, is a good alternative and has been enthusiastically introduced in the SELEX process [68,69].

#### 2.2.4. Sequencing

In general, specific aptamers are identified in the last round of selection by the traditional Sanger-sequencing method. However, this method may not reflect the real situation of aptamer enrichment. For example, only few sequences are identified, and this limited sequence information may not be enough to identify true binding sequences. Another shortcoming is that some important binding sequences present in previous rounds may be lost. Taking this issue into an account, high-throughput sequencing technology, whose decreasing costs have become acceptable to many laboratories now, is increasingly used to identify high-affinity binding aptamers in pre-terminal selection rounds during the SELEX process [21,69,70,71]. This approach can globally monitor the true aptamer enrichment situation and track the mutation rate in aptamer evolution. Despite the advantages of high-throughput sequencing, the lack of adequate computational approaches makes it difficult to analyze the copious amount of data generated in each selection round (2–50 million sequences). Fortunately, Hoinka *et al*. reported a new computational algorithm to close this gap [72,73] by analyzing mutant information and simultaneously clustering related aptamer sequences [74].

In summary, to achieve successful and efficiently-developed SELEX products is no longer a difficult task. With careful design of the initial oligonucleotide pool, efficient partitioning, accurate amplification, global analysis of sequencing data, and in-time adjustment of the selection parameters in the SELEX process, the success rate can be improved significantly. To expand the applicability of aptamers, some interesting strategies have also been reported recently, such as cocktail aptamers and epitope-specific SELEX. Some aptamers obtained previously have suboptimal affinity against their target molecules, but paired aptamers bound with non-overlapping epitopes on the same target molecule can be used to form aptamer cocktails with significantly improved affinity and specificity. Indeed, aptamer cocktails improved target binding affinity 210–390 fold [75,76]. However, most SELEX processes are considered to operate within a black box in that the particular binding sites of obtained aptamers are not known in advance. To overcome this problem, Lao *et al*. developed epitope-specific SELEX that can select aptamers that bind to a desired epitope [77]. This technique makes possible the selection of aptamers with predicted functions. Another developmental direction of SELEX technology is high-throughput and automated selection. In a particular example, a Quantitative Parallel Aptamer Selection System (QPASS) was developed that integrated microfluidic selection, high-throughput sequencing, and *in situ*-synthesized aptamer arrays [78]. QPASS can simultaneously measure the affinity and specificity for thousands of candidate sequences in parallel.

## 3. Improvement in Applications

Due to their functional similarity with antibodies, aptamers have been used extensively in various biomedical applications that were once the sole realm of monoclonal antibodies, including as research tools [79], in bioassays [80,81], for cell detection [82,83,84,85,86,87], tissue staining [88,89], *in vitro* and *in vivo* imaging [90,91,92,93,94], targeted therapy and nanomedicine [95,96,97,98], and food safety and environment monitoring [99,100,101,102]. Recently, a plethora of reviews have been published by groups, including ours, that discuss the various applications of aptamers, such as disease diagnosis and therapy [10,11,28,103,104,105,106], bioassays [3,107], and food safety and environment monitoring [108,109]. This review focuses instead on the applications in which aptamers perform differently or better than monoclonal antibodies. For example, (i) aptamers and Cell-SELEX technology can be used as biomarker discovery tools; (ii) aptamers can be used as more sensitive and safer bioimaging agents; (iii) aptamers with switchable conformation can be used to develop precisely controlled target-triggered assays or drug release systems; and (iv) aptamers are easily uploaded with chemical or gene therapy drugs for targeted delivery.

Novel biomarker discovery is urgent needed in the personalized cancer research field. To this aim, Cell-SELEX can be used as a powerful tool for novel biomarker discovery without prior knowledge of biomarkers present on tumor cell surfaces. By using specific cancer cells as targets and normal/noncancerous cells as negative-selection targets, aptamers specifically against the unique cancer cells will be obtained. After target affinity purification and mass spectrometry analysis, the novel biomarker(s) will be identified. Some novel biomarkers with different expression levels on cancer cells have been discovered through this method, including protein tyrosine kinase-7 [16,110] and immunoglobulin mu heavy chain [111,112]. However, affinity purification and mass spectrometry analysis are not always effective because some membrane proteins are difficult to isolate by affinity purification due to their high hydrophobicity and low solubility. Taking this issue into account, some bioinformatics-based strategies have been introduced recently, such as comparison analysis of correlations between protein expression levels and aptamer binding intensity in different cells [113] and online bioinformatic software analyses [114]. Different from this type of “single” biomarker discovery to specific disease, combinatorial ligands library based-proteomics technologies are more powerful tools to identify a set of disease-relative biomarkers simultaneously, such as multiplexed antibodies- [115], combinatorial peptide ligands- [116] and oligonucleotide aptamers-based assays [117], which have been deeply used to screen known or unknown biomarkers.

**Figure 4:**
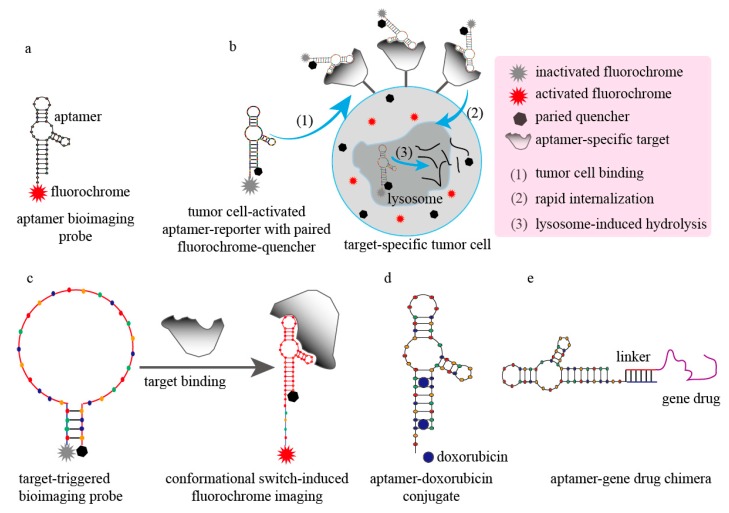
Some representative models of modified aptamers for disease diagnosis and therapy. (**a**) An aptamer labeled with a fluorochrome is used as a sensitive bioimaging probe; (**b**) Tumor cell-activated aptamer-reporter with paired fluorochrome-quencher molecules are used in rapid, one-step, high-throughput assays for circulating tumor cells within drops of blood; (**c**) Switchable aptamer-based fluorochrome-quencher reporter are used as precisely controlled, target-triggered, bioimaging probes; (**d**) The simple, but effective, aptamer-doxorubicin conjugates; (**e**) Aptamer-gene drug chimera used for targeted delivery of gene-silencing tools.

Small oligonucleotide aptamers have clear superiority over antibodies for *in vitro* or *in vivo* bioimaging applications. Aptamers have higher sensitivity and improved safety *versus* antibody-based bioimaging agents, and benefit from the properties of easy tissue penetration and rapid renal clearance rates. Conversely, antibody-based bioimaging agents suffer from poor tissue penetration, high immunogenicity, and long blood residence time that can result in unwanted side effects [118]. Our group developed two kinds of CD30 aptamers, RNA and DNA versions of the IRD800CW reporter for specifically imaging CD30-positive lymphoma tumors *in vivo* (Figure 4a) [92]. Our results show that injected CD30 aptamer-reporters rapidly accumulate (<10 min) into CD30-positive tumor sites, but not into CD30-negative tumor sites, with the imaging signal intensity 4–8-fold higher than in control tumors and body background levels. The imaging signal of CD30 RNA-based aptamer reporters is stable for up to 60 min, but DNA-based aptamer-reporters maintain stability for up to 24 h. Minimal aptamer accumulation was detected in normal tissues [92]. In a related experiment, ^111^In-labeled anti-human epidermal growth factor receptor antibody or aptamer was conjugated with hollow gold nanospheres for head and neck cancer *in vivo* imaging. The tumor uptake of aptamer-guided imaging probes was much higher than that of the antibody-guided imaging probes [94].

However, this type of aptamer or antibody-guided reporter system is considered to be a signal “always-on” probe, which results in high background noise. To overcome this obstacle and further improve targeting sensitivity, our group developed a cancer cell-activated aptamer-reporter system for one-step assaying of circulating tumor cells (Figure 4b) [119]. Through our design, the paired fluorochrome and quencher molecules are respectively attached to the 5′- and 3′-terminal ends of biomarker-specific aptamers. Due to close proximity, the fluorochrome is quenched by the paired quencher molecule. In the absence of target-specific cells, the intact aptamer-reporter system is optically silent. When the aptamer-reporter system interacts with target-specific cells, the aptamer-reporter system is internalized into cell lysosomes and degraded rapidly due to the high nuclease sensitivity of aptamers. Thus, the fluorochrome is separated from the quencher and emits a bright fluorescent signal within minutes, exclusively from inside the target-specific cancer cells and without any background noise. By using an anti-CD30 aptamer that is labeled with paired Cy3 fluorochrome and Black Hole Quencher 2 (Life Technologies Inc., Carlsbad, CA, USA) as a model, when CD30-positive cancer cells were present, the Cy3 fluorescent signal was activated within 10 minutes and was stable for up to 120 min with undetectable background noise. This simple, but highly sensitive, aptamer-reporter system can be used to generate a rapid (<1 h) one-step high-throughput assay for identifying circulating tumor cells within drops of blood [119].

Because aptamer conformations are switchable when they bind/unbind to their targets, aptamers can be used to develop precisely controlled target-triggered assays or drug release systems that can significantly reduce background noise and side effects. For example, the TD05 aptamer was modified to create a switchable structure by adding a single-stranded DNA complementary extension, and then further modified by attaching the fluorophore AlexaFluor488 and the quencher Black Hole Quencher 1 (Life Technologies Inc.) at the respective 5′- and 3′-terminal ends. In principle, when the target-specific B-cell lymphoma cells are absent, the unbound TD05 aptamer-reporters remain optically silent. When B-cell lymphoma cells are present, TD05 aptamer binding to the cell-surface targets changes the TD05 aptamer-reporter conformation. Thus, the fluorophore is activated because the suppressing quencher is now out of reach (Figure 4c) [120]. In experiments, the optical signals of this switchable TD05 aptamer-reporter increased by eight-fold within 15 min of coincubation with the specific target cells [120]. Based on this type of conformational-switchable aptamer model, some target-triggered drug-release systems have been recently developed. For example, switchable anti-thrombin aptamers labeled with Ce6 photosensitizer were conjugated with single-walled carbon nanotubes for thrombin-triggered photodynamic therapy [121], and switchable anti-adenosine triphosphate aptamers were hybridized with mesoporous silica nanoparticles for adenosine triphosphate-triggered drug delivery and release. Regado Biosciences Inc. (Basking Ridge, NJ, USA) (now merged with Tobira Therapeutics Inc., South San Francisco, CA, USA, in May 2015) developed an aptamer-antidote system (REG1) comprised of a 37-mer RNA aptamer against factor IXa and a 17-mer complementary sequence for precisely controlled coagulation therapy [122] that has passed phase II clinical trials [123]. However, based on a recommendation from the trial’s Data and Safety Monitoring Board (DSMB) that indicated the level of server allergic adverse events associated with REG1 system was of frequency and severity, Regado Biosciences Inc. terminated the enrollments of participants for phase III clinical trial recently.

Another application advantage is that aptamers are easily uploaded with small chemicals, especially gene-therapy drugs. A simple, but effective, strategy is to non-covalently intercalate small chemical drugs, such as doxorubicin, into aptamer three-dimensional structures at juxtaposed GC/CG pairing sites (Figure 4d). A series of studies have shown the effectiveness of this simple aptamer-doxorubicin conjugation that results in improved therapeutic indices and decreased side effects when using epithelial cell adhesion molecule aptamer-doxorubicin conjugates for retinoblastoma-targeted therapy [124], HER2 aptamer-doxorubicin conjugates for breast cancer-directed therapy [15], MUC1 aptamer-doxorubicin conjugates for lung cancer-targeted therapy [125], and prostate-specific membrane antigen aptamer-doxorubicin conjugates for prostate cancer-specific therapy [126].

Gene therapeutic techniques and drugs, such as RNAi and DNAzyme, constitute a class of powerful gene-silencing tools, but their lack of cell/tissue specificity results in unwanted off-target effects. Due to the nature of nucleic acids, gene-therapy drugs are easily conjugated with aptamers to form aptamer-gene therapeutic drug chimeric structures that achieve targeted therapy (Figure 4e). Subramanian *et al*., developed an anti-nucleolin aptamer-survivin DNAzyme chimera by hybridizing the aptamer with DNAzyme through a poly-A:poly-T linker. The resultant aptamer-DNAzyme chimera specifically targeted nucleolin-positive cancer cells, was internalized efficiently, and cause significant cell death [127]. Recently, other types of aptamer-RNAi chimeras have also achieved the desired outcomes. These include aptamer-siRNA chimeras [128,129], aptamer-miRNA chimeras [130,131], and aptamer-shRNA chimeras [132].

## 4. Conclusions

Aptamer and SELEX technology have brought a revolution to biomedical fields since their introduction in 1990. Although monoclonal antibody-based products still dominate the markets of targeted diagnosis and therapy, the high immunogenicity and production costs of antibodies remain the main challenges that limit their clinical application. As small, multifunctional, oligonucleotide ligands, aptamers show immense potential to be excellent alternatives or supplements to monoclonal antibodies. Aptamer research greatly overlaps with applications using monoclonal antibodies, and in some research fields, aptamers have shown superiority over monoclonal antibodies, including as biomarker discovery, *in vitro* and *in vivo* diagnosis, and precisely controlled targeted therapy. Although successful commercialization of aptamer-based drugs remains a few years away, research scientists, including our group, and pharmaceutical companies have great confidence and enthusiasm in their utility. A commercial report published by MarketResearch.com (Rockville, MD, USA) predicted that the global aptamer market, including therapeutics, diagnostics, biosensors, drug discovery, biomarker discovery, and research applications, would reach $5.4 billion by 2019 [133].

As outlined earlier, the main impediments to aptamer development and application are their suboptimal bioavailability and shortcomings in the SELEX process. However, the technical barrier of limited bioavailability of aptamers has been successfully solved using different chemical and structural modifications. Thus, improving the success rate and shortening the selection period in the SELEX process have become the most important issues. Fortunately, some improved strategies have achieved these desired outcomes, such as SOMAmer, bead-based selection, Cell-SELEX, and microfluidics technology.

Despite the tremendous translational potential for aptamers in clinical applications, there are not yet enough useful data from clinical trials, and most aptamer-based products remain in preclinical pipelines. As a novel class of targeted ligands, the effectiveness and safety of aptamer-based drugs should be carefully evaluated in more preclinical and clinical trials. As more research scientists and pharmaceutical companies realize the important application potential of this type of small nucleic acid molecule, aptamer-based products will begin entering markets in the near future.
